# A169 A RARE MANIFESTATION OF ENTERITIS DUE TO IMMUNE CHECKPOINT INHIBITOR: CASE REPORT OF PYLORIC STRICTURE

**DOI:** 10.1093/jcag/gwad061.169

**Published:** 2024-02-14

**Authors:** A Cintosun, N Bollegala

**Affiliations:** University of Toronto, Toronto, ON, Canada; University of Toronto, Toronto, ON, Canada

## Abstract

**Background:**

Immune checkpoint inhibitors (ICI) are immunomodulatory antibodies used to enhance the immune response against tumor cells that have revolutionized the treatment of various malignancies. ICIs pose a risk of immune-related adverse events in various organs including the gastrointestinal tract, with diarrhea and colitis being most common. ICIs can rarely cause involvement of the upper gastrointestinal tract, with case reports of gastritis, duodenitis, and esophagitis. There are very rare reports of strictures caused by ICIs, more frequently in the lower gastrointestinal tract, and only a few reports in the esophagus or duodenum.

**Aims:**

We report a rare case of an upper gastrointestinal stricture due to ICI at the pylorus.

**Methods:**

Case report.

**Results:**

A 38-year-old woman with breast cancer on ICI pembrolizumab presented with several weeks of nausea and early satiety. She had started pembrolizumab 8 months prior, underwent neoadjuvant chemotherapy and mastectomy months ago, and completed radiation 6 weeks earlier. She also described nocturnal reflux and heartburn, but no vomiting, weight loss, or other gastrointestinal symptoms. Her examination was normal and recent bloodwork only showed a mild normocytic anemia.

EGD (esophagogastroduodenoscopy) showed friable circumferential ulceration of the distal antrum with surrounding erythema and luminal narrowing from the pyloric channel to duodenum. 1-centimetre gastroscope was not passable but 4.9-millimetre pediatric gastroscope passed into the duodenum where there were no abnormalities past the duodenal bulb. Pathology showed granulation tissue, fibrinopurulent exudate, and antral mucosa with chronic active inflammation negative for Helicobacter pylori and malignancy. Oncology felt that radiation was too far from the pylorus to be responsible for the findings.

High-dose proton pump inhibitor (PPI) and soft diet were recommended with minimal symptomatic improvement. 2 months later, she developed weight loss and repeat EGD showed minimal endoscopic improvement (Figure 1).

ICI-related enteritis was suspected and she was started on prednisone 50 mg daily. Her appetite and nausea significantly improved. Steroid taper was planned for 8 weeks.

At 3 months, symptoms recurred with prednisone taper. EGD showed only mild improvement of the duodenal stricture. Prednisone dose was increased. On repeat EGD at 7 months, the pylorus was patent with only granular inflammation predominantly in the apex of the duodenal bulb. She tolerated a slower prednisone taper thereafter.

**Conclusions:**

ICI-related inflammation is common in the lower gastrointestinal tract and can less commonly affect the upper gastrointestinal tract. This is a rare report of upper gastrointestinal stricture and the first report known to us of pyloric stricture caused by ICI. Treatment may be challenging, but was achieved with PPI and high-dose steroids with slow taper.

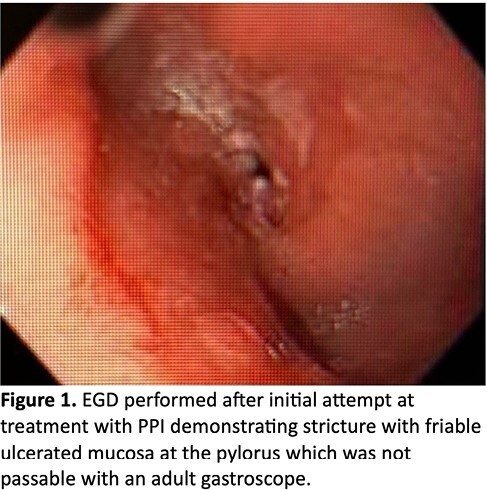

**Funding Agencies:**

None

